# Perception of caring behaviors and associated factors among nurses working in public hospitals of East Wollega Zone, Ethiopia, 2023. Mixed method study

**DOI:** 10.3389/fpubh.2025.1513819

**Published:** 2025-04-02

**Authors:** Takele Mitiku Tesema, Eba Abdisa Golja, Lammi Atomsa, Yohannes Midekso Berisa

**Affiliations:** Department of Nursing, School of Nursing and Mid-wifery, Institute of Health Sciences, Wallaga University, Nekemte, Oromia, Ethiopia

**Keywords:** caring behaviors, hospitals, mixed method, nursing care, perception

## Abstract

**Background:**

The perception of nurse caring behaviors significantly impacts the patient's satisfaction, trust, and health care experiences. Good perception is associated with positive outcomes. Nursing research has examined nurse's perception of caring behaviors and research shows low perceptions of caring behaviors globally. In Ethiopia, limited research exists on nurses' perceptions of caring behaviors and related aspects of the nurses. However, further investigation including a mixed-methods study is needed to explore this topic and identify factors which affects nurse's perception of caring behaviors.

**Objective:**

To assess nurses' perception toward caring behaviors and associated factors among nurses working in Public Hospitals of East Wallaga Zone, 2023.

**Methods:**

A Facility-based Convergent mixed-method study was conducted from May 30 to July 30, 2023. Among Nurses in East Wollega Zone Public Hospitals, 394 study participants were selected by Simple random sampling technique for quantitative data and six (6) study participants were used for the qualitative data collection based on information saturation. Quantitative data was entered into Epi-data version 3.02 and analyzed by SPSS Version 25. In bi-variable analysis, candidate variables were selected for multivariable analysis model at a *P* < 0.25. In multi-variable logistic regression analysis, adjusted odds ratio (AOR) with 95% CI was used to assess the association of independent variables on the perception of nurse toward nursing care behavior. A *P* < 0.05 was considered statistically significant. For qualitative data, narrative thematic analysis was used.

**Result:**

The percentage of good perception toward nurse caring behaviors was found to be 60.9% (95% CI: 55.7–65.85). Being single [AOR: 3.90, 95% CI (1.19–12.83)], work experience [AO: 0.43, 95% CI (0.19–0.97)], Professional Satisfaction [AOR: 3.3, 95% CI (1.96–5.84)], Having bad relationship with staff [AOR: 0.04, 95% CI, (1.21–3.45)], and Job satisfaction [AOR: 2.4, 95% CI (1.46–4.17)] were found to be significant factors associated with nurses perception toward nurse caring behaviors in the study setting.

**Conclusion and recommendation:**

Findings of this study revealed that the perception of good caring behaviors among nurses was found to be low relative to literature. Therefore, Hospital management, Nurse Directors and Health bureaus need to create harmonized work relations and to motivate nurses need to pay attention to quality nurse care and improve perception of nurses on caring behaviors.

## Background

Nursing is caring centered, which sets nurses apart from other healthcare workers and is regarded as the essence of humanistic clinical nursing practice. To raise the standard of healthcare, nurses are urged to provide the best care possible. According to Watson's Theory of Human Caring, the nurse-patient relationship is what makes caring strong, and it helps the care recipient preserve their dignity and achieve holistic health and nurses form genuine transpersonal loving relationships with patients (1).

Nurses' perceptions of caring behavior are defined as “nurse perceptions of their activities, behavior, and mannerisms that have a great concern to make and preserve a trust relationship with the patient, pay attention to the patient, and to safeguard the patients in hospital” (2). It is used to describe how nurses perceive their behavior as being caring. It involves treating patients with respect, promoting safety and reducing anxiety, engaging in therapeutic conversation, displaying professional knowledge and skills, and paying attention to the patients (3). The perception of nurse caring behaviors significantly impacts the patient's satisfaction, trust and health care experiences then good perception is associated with positive outcome during care.

The perception of caring, as well as individual differences, can have a direct effect on work behaviors. People with varied perceptions have different characteristics, needs, world views and interactions with supervisors, co-workers, subordinates and customers (4).

The provision of high-quality care and services has been highlighted in the healthcare system, particularly in the area of nursing services (5, 6). The purpose of nursing is “human health care,” and “care” is one of the core and well-known nursing practices (7, 8). Studies have shown that patients' and nurses' perspectives of care can vary, particularly when patients and nurses come from different national or cultural backgrounds and have different perspectives on the same concepts (9) .

Clarifying the factors that may influence nurses' perceptions of caring behaviors remains a highly important issue for the international nursing community. On a Saudi multicultural level, understanding caring behaviors presents a major challenge for nurse clinicians, educators, and researchers (10). According to evidences from studies nurse's perception of caring behavior on the part of other nurses has been linked to a variety of contributing factors had an impact on perception of caring behaviors (11–14).

Globally, 49.3% of patients were unsatisfied and 7.8% of patients in Asia who had national health insurance were extremely dissatisfied with the hospital services due to poor perception of caring behaviors (15), a study conducted in Iran on a sample of nurses, the average perception of caring behaviors among nurses in Iran was 81.8% which declare related factors are high impact on perception of nurses caring behaviors (16).

Studies in Ethiopia show that nurses' perceptions of caring behaviors vary across regions and healthcare settings, in Southern Ethiopia, 75.1% of nurses reported good perceptions in maternity care wards (17) while 80.3% in Jimma University Specialized Hospital (18). In Harar and northern Ethiopian hospitals, 63.4 and 68.2% of nurses have a positive opinion of caring behaviors, suggesting suboptimal perceptions among nurses, indicating poor outcomes in caring (11, 12). However, in Harari region hospitals, 51.67% had poor caring behaviors, suggesting suboptimal perceptions and poor outcomes for nurse professionals (19). According to the data from findings, fewer nurses were found to have a good perception of caring behaviors. Based on the available data the perception of nurses in Ethiopia may have a suboptimal perception of caring behaviors. This funding's suggests nurse professionals are poor outcome on caring.

According to previous studies nurse's perception of caring behavior on the part of other nurses has been linked to a variety of sociodemographic traits, those contributing factors had an impact on perception of caring behaviors (11–14).

Through the implementation of hospital reform guidelines, the Ethiopian Federal Ministry of Health is working to increase the quality and accessibility of healthcare services; one of the key priorities of this guideline is enhancing the quality of nursing care (20).

To evaluate and enhance the quality of care, nursing research has excessively examined nurses' assessment and perception of caring behaviors and underlying factors in various clinical care settings worldwide. However, there is lack of adequate information about perception of caring behaviors. The assessment of a nurse's caring behavior is then influenced by a range of factors, and multiple studies have revealed that nurses have low expectations for caring. But still further research must be needed in Ethiopia on aspect of nurses' perceptions of caring behaviors and associated characteristics of the nurses who provide care services. Consequently, it is necessary to look into additional elements that might influence how nurses perceive caring behaviors. To understand how nurses are perceived as being the perception of caring behaviors, a mixed-methods study is crucial. Therefore, the current study aimed to assess the perception of caring behaviors and associated factors among nurses working in East Wollega Zone public Hospitals, in western Ethiopia.

**Figure 1:**
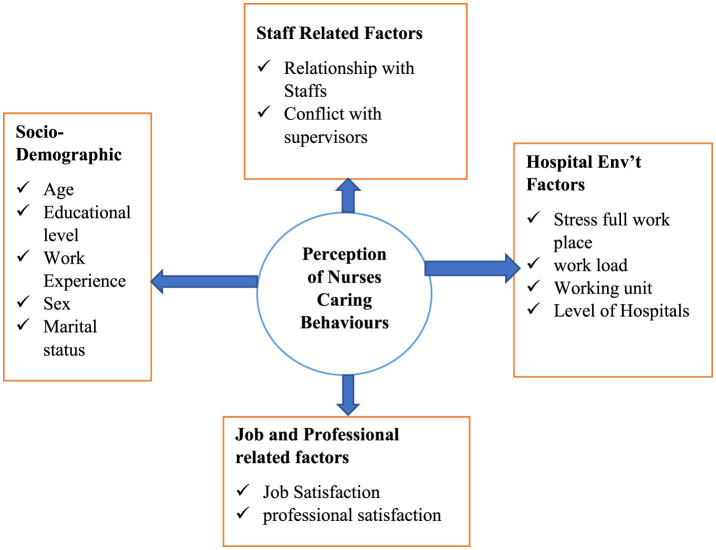
Conceptual frame work adapted to assess nurses' perception toward nurse caring behaviors and associated factors.

The significance of this study lies its potential to the gaps in perception of nurses caring behaviors and its impact on practice. The findings of the study can serve as improve practice and ultimately valuable embrace satisfaction for nurses enabling them to the patients benefit from these findings when the nurse's perception of caring behaviors is improved. The positive impact of improving nurse's perception of caring behaviors is for teaching also for patients. Furthermore, this findings is holds relevance for policymakers and organizations that was to inform decision making on high-quality care to the patients (Figure 1).

## Methods and materials

### Study design

A mixed method study design was carried out, which was Convergent or parallel studied, both quantitative and qualitative. For quantitative, a facility-based cross-sectional study design was employed to assess nurses' perception of caring behaviors and associated factors. For qualitative phenomenological Convergent study design was used to explore the experience of nurses' perception of nurse caring behaviors at study site.

### Study area, study population and eligibility criteria

The study was conducted in public hospitals of East Wallaga Zone from May 30 to July 30. Currently, East Wollega Zone has five public Hospitals and one private Hospital, 17 Woreda Health Offices, 58 Health Centers, and 287 Health Posts. All nurses who are employed in East Wollega zone public hospitals. All sampled nurses who fulfill inclusion criteria and available during data collection period. Individual nurses who fulfill inclusion criteria and available during data collection period Nurses who were employed in Hospitals of East Wollega Zone and volunteer to participate in the study, were included in the study. Nurses who were on leave (Annual, Maternal, etc.) during data collection were excluded from the study and nurse who were critically ill. For the qualitative data six (6) study participants were used based on information saturation, it means if the responses of our participants redundancy raised focus on the same question the data reached our findings.

### Sampling procedure

All hospitals in the East Wollega Zone was covered by this investigation. For every hospital, a proportionate sample size allocation was used for the purpose of getting equal chance selection for each hospital based on our total sample size. The study participants were chosen by simple random sampling from the list of nurses who met the inclusion criteria. The sampling frame was created using the nurse lists that were acquired from each hospital. Additionally, the purposive sample strategy was employed to choose study participants for the qualitative study.

### Data collection instruments

#### For quantitative

The quantitative data was collected through a self-administered questionnaire. The outcome variable (nurse perception about their caring behavior) was assessed by the latest revised version of the Nurse caring behaviors Inventory (CBI-16) internationally accepted and standardized self-administered questionnaire and validated for Ethiopian health care providers and adopted one (21, 22). The general questionnaire of this study has three parts: socio-economic factors, associated factors related questions, and Perception of Nurse Caring Behaviors. The CBI-16 measures four dimensions of nurse caring: Assurance (five items), Knowledge and skill (four items), Respectfulness (four items) and Connectedness (three items). It was designed and validated for administration to nurses, rating the frequency of caring behaviors on a 6-point Likert scale from 1 “never” to 6 “always” (21, 22). The internal consistency of the instrument was checked using Cronbach's alpha (α = 0.935). Evaluation of the scale is done based on the total scores and each dimension's scores. Thus, a low score below mean indicates a low perception of Nurse caring behaviors and a high score above mean indicates a high perception of Nurse caring behaviors (21, 22).

Job satisfaction was assessed by 15 items and each item has a 5-point Likert-type scale (very dissatisfied, dissatisfied, neutral, satisfied, and very satisfied) developed by Warr et al. (36). The internal consistency of the instrument was checked using Cronbach's alpha (α = 0.896). The overall job satisfaction score was estimated by taking the average score of all the subscales (23). Nurse–Staff relationships and conflict with supervisors was assessed by the Nurse–Staff Collaboration Scale item, which consist of 23 items (24). The internal consistency of the instrument was checked using Cronbach's alpha (α = 0.985). Workplace stress full environment was assessed by Occupational stress scale: A three-point Likert scale was used where stress low = 1, moderate stress = 2, and extreme stress = 3. The total number of statements included in the scale was 15; whereas, the total mean score was calculated by summating all statements for every nurse and then dividing by the total (25). Work load was assessed by 15 items and each item has a 5-point Likert-type scale (26). The internal consistency of the instrument was checked using Cronbach's alpha (α = 0.934). Professional satisfaction was assessed by 12 items and each item has a 4-point response scale (strongly disagree to strongly agree) (27). The internal consistency of the instrument was checked using Cronbach's alpha (α = 0.745).

#### For qualitative study

The qualitative data collection was conducted by using semi-structured Afan oromo interview guide questions, which were primarily prepared in English by the principal investigator based on previous studies and then translated into Afan Oromo by a fluent speaker of the language. The participants were purposively selected to share their experiences and thoughts related to the phenomena. The interview took place in a quite private room. The interview was facilitated by the investigator, and the mean average time taken for the interview was 15 min. The interviewer took mobile phone records and field notes.

### Data collection procedures

Before data collection, Five BSc Nurses as data collectors and one MSc Nurses as supervisors were recruited and given 1 day of training on the content of questionnaires and procedures of data collection. The questionnaires was distributed after asking the permission from the institution and participants then the participants answered each question accordingly, and checked the questionnaires and they gave them to the investigator. A pretest on 5% (24 participants) of the sample was conducted at Bedelle General Hospital and ambiguous questions was clarified and corrected. During data collection, close supervision was executed and daily checkups of filled questionnaires on completeness and appropriateness was done by supervisors and the investigator.

A pretest was carried out on 5% of the sample size in Bedelle General Hospital. During the data collection period, appropriate supervision was done, and daily checkups of the questionnaire for completeness were affected. Above all, ethics, coding, and entry were maintained throughout the process. All documents were kept properly, and the computer version of the data was also protected by a password, which was accessed only by the researcher.

### Ethical considerations

Ethical clearance was obtained from the Wallaga University Institute of Health Science, School of Nursing and Midwifery, and the research ethical review committee on **Date May 2023, (Minutes No: 1028/2023)**. Hospitals were communicated legally for their permission and each of the interviewees was asked for their informed written and signed voluntary consent form before answering the question. Before the self-administered questionnaire and recording telephone interview, the objective of the study was verbally clarified for each participant and the participants' questions were answered. The confidentiality of the study participants was maintained during the distribution and data collection periods and assured by not recording the interviewee's name on the questionnaire.

### Measures to confirm trustworthiness of qualitative data

Trustworthiness is a measure of the quality of the research, which entails, Credibility, Transferability, dependability, and confirmability. The researcher ensured the credibility of the research through immersion in the field and by using a variety of strategies to collect data, which included interviews, observation, field notes, and reflexive journals. And the interviewer was openly communicating with participants to tell true or credible information about their experience. The researcher presented the strategies and corrections were made based on the recommendations of experts and advisors in research. The researcher ensured transferability by providing a rich description of the demographic of the participants and a description of findings in such a manner that another person can compare it with the findings of other studies. Dependability was ensured by providing a dense description of the research methodology, supported by a literature review and to ensure the dependability of the study information about data collection, time, place, and analysis was provided. Confirmability is a criterion for trustworthiness in a qualitative inquiry, which refers to the neutrality of the data and interpretations. To ensure confirmability, the entire research process was audited by the study supervisors, advisors, and independent coders who worked closely with the researcher, and the study description was supported by quotations.

### Study variables

#### Dependent variable

- Nurse's Perception of caring behavior.

#### Independent variables

- **Socio-demographic factors**: Age, sex, marital status, residence, educational level, and work experience.
- **Hospital environment factors**: Workload, type of institution, working unit, and stress full work place.
- **Staff-related factors**: Relation with staff, conflict with a supervisor, job satisfaction and satisfaction with their profession.

#### Operational definition

- **Good perceptions:** For this study, nurse workers whose scores were above the mean (≥75 score) were considered as having good perception (11, 12).
- **Job satisfaction:** For this research, nurse workers whose score below the mean (< 49 score) were considered dissatisfied, and those with a mean above (≥49 score) were regarded as satisfied (11).
- **Professional satisfaction:** For this study, nurse workers whose score below the mean (< 33 score) were considered poor satisfaction, and those with a mean above (≥33 score) were regarded as good satisfaction (11)
- **Workload:** For this research, nurse workers whose score below the mean (< 47 score) were considered low work load, and those with a mean above (≥47 score) were regarded as high work load (11, 12).

### Data processing and analysis

Data entry and analysis were done using Epi-Data version 3.02, and exported to SPSS version 25+ computer software. Nurses' perception of caring behaviors was presented For each subgroup score and total CBI-16 score. Categorical variables were summarized as numbers and percentages. To measure the association between the outcome and independent variables, an adjusted odds ratio (AOR) along with a 95% confidence interval was calculated. To identify factors associated with the outcome variable; first, a binary logistic regression analysis was performed for one independent variable with the outcome variable. Then, variables with a *p*-value < 0.25 were considered for the multivariable logistic regression model to control confounders. Normality test was conducted to identify whether the data was normally distributed or not and the data was normally distributed. Multi-collinearity test was done with a variance inflation factor (VIF). Model goodness-of-fit was tested by Hosmer-Lemeshow. Backward Stepwise (Likelihood Ratio) method of variable selection was used. Statistical significance was declared for variables with *p*-value < 0.05 with 95% CI.

### Qualitative data analysis

For qualitative, data analysis was performed by using thematic analysis. An in-depth audio-recorded interview was changed to text and checked for consistency and entered ATLAS-ti 8.14 software for analysis and grouped into themes then finally the result of qualitative data was presented in narration based on interview findings. Due to phenomenological Convergent study design was used we collect the exploration of nurses' experience on perception of nurse caring behaviors at study site and presented in narration under theme organized.

## Results

### Quantitative and qualitative result

A total of 394 nurses were enrolled after fulfilling the inclusion criteria. Complete data were obtained from 373 with response rate of (94.6%) participants was participated in this study from primary, general, referral, and specialized hospitals. From the enrolled participants 21 of them did not fill the questionnaires fully which makes the non-response rate 5.3% because of Self-administrated questionnaires.

### Socio-demographic characteristics of the respondents

Most of the study participants 176 (47.2%) were found in the age category of 25–30 years and mean age of the respondents with (Mean = 31.27). One hundred ninety-seven (52.8%) of nurses were males. Two hundred twenty-one (59.2%) of the nurses were married. The result also depicted that 328 (87.9%) of nurses' educational level were degrees whereas 11 (2.9%) of nurse's educational level were master's holder. In this study the majority 117 (31.4%) of nurses were working in Specialized Hospital while 108 (29%) of nurses were working in Primary Hospitals and Referral hospital 100 (26.8%). The result also showed that 190 (50.9%) of nurses said that they have satisfied (Tables 1, 2).

**Table 1:** Socio-demographic characteristics of participants in EWZ Public Hospitals, Oromia, Ethiopia, 2023.

**Variable**	**Category**	**Frequency**	**Percent**
Age *N* = 373	20–25	44	11.8%
	26–30	176	47.2%
	31–35	84	22.5%
	36–40	40	10.7%
	>40	29	7.8%
Sex *N* = 373	Male	197	52.8%
	Female	176	47.2%
Marital status *N* = 373	Married	221	59.2%
	Single	127	34.0%
	Widowed/ divorced	25	6.7%
Religion *N* = 373	Protestant	253	67.8%
	Orthodox	78	20.9%
	Muslim	34	9.1%
	Wakefata	6	1.6%
	Others^*^	2	0.5%
Educational level	Diploma	34	9.1%
	Degree	328	87.9%
	Masters	11	2.9%
Work experience	1–5	167	44.8%
	6–10	142	38.1%
	>10	64	17.2%

Others^*^ “Jevoa” “Seventh Day Adventist”.

**Table 2:** Characteristics of participants with their work place in EWZ Public Hospitals, Oromia, Ethiopia, 2023.

**Variable**	**Category**	**Frequency**	**Percent**
Hospital type	Primary	108	29.0%
	General	48	12.9%
	Referral	100	26.8%
	Specialized	117	31.4%
Working unit	Medical	70	18.8%
	Surgical & OR	79	21.2%
	Pediatrics	60	16.1%
	Emergency	47	12.6%
	OPD [TB, NCD, ART]	73	19.6%
	AICU/NICU	26	7.0%
	Orthopedic	8	2.1%
	Oncology	10	2.7%
Job satisfaction	Satisfied	190	50.9%
	Dissatisfied	183	49.1%
Conflict with supervisors	No conflict	191	51.2%
	Conflict	182	48.8%
Nurse-staff relationship	Good relationship	179	48.0%
	Poor relationship	194	52.0%
Stressful work place	No stress	208	55.8%
	Stressful	165	44.2%
Work load	Low workload	211	56.6%
	High workload	162	43.4%
Professional satisfaction	Good satisfaction	190	50.9%
	Poor satisfaction	183	49.1%

A total of six Nurse Professionals participated in the qualitative study. Before the telephone interviews, participants were requested to provide their demographic data which included age, sex, work experience, and language. There were five male participants and one female. Regarding language six of the participants were Afan Oromo speakers which was used for the interview. Other Information mentioned below (Table 3).

**Table 3:** Socio-demographic characteristics on qualitative data of nurse perception about nurse caring behaviors in public hospitals of east Wollega zone, Oromia, Ethiopia, 2023.

**Participants**	**Age**	**Sex**	**Level of education**	**Total work experience**
P1	37	M	MSc	12
P2	30	M	MSc	10
P3	25	M	BSc	4
P4	25	M	BSc	2
P5	34	M	BSc	13.5
P6	29	F	BSc	6

#### Nurse caring behaviors in subscales and total score

The result of this study revealed that nurses' perception of nurse caring behaviors in assurance and knowledge subscales were found to be mean of 4.62 and 4.78, respectively. Nurses' perceptions of nurse caring behaviors in respectfulness and positive connectedness subscales were 4.79 and 4.77, respectively. The total CBI-16 score of nurses' perception of nurse caring behaviors was found to be mean of 4.74 (Table 4).

**Table 4:** Assurance of human presence, knowledge and skill, respectfulness and connectedness with subscale scores and total of CBI-16 subscales questions for participants in East Wallaga Zone Public Hospitals, Oromia, Ethiopia, 2023.

**Subscales**	**Questions**	**Mean ±SD**
Assurance of human presence	Returning to the patients voluntarily	4.24 ± 1.365
	Talking with the patients	4.61 ± 1.275
	Responding quickly to the patient's call	4.62 ± 1.272
	Give the patients' treatment and Medication on time	4.91 ± 1.278
	Relieve patients symptoms	4.75 ± 1.247
	Total score of assurance of human presence	4.62 ± 1.287
Knowledge and skill	Being confident with patients	4.84 ± 1.264
	Demonstrating professional knowledge and skill	4.79 ± 1.277
	Treating patient's information confidentiality	4.98 ± 1.183
	Meeting patient's stated and unstated needs	4.51 ± 1.335
	Total score of knowledge and skill	4.78 ± 1.26
Respectful deference to others	Attentively listening to the patients	4.84 ± 1.258
	Treating the patients as individual	4.64 ± 1.409
	Supporting the patients	4.98 ± 1.255
	Being empathetic with the patients	4.70 ± 1.362
	The total score of respectful deference to others	4.79 ± 1.321
Positive connectedness	Instructing patients	4.79 ± 1.207
	Spend time with patients	4.72 ± 1.257
	Include the patients in the planning	4.82 ± 1.235
	Total score of positive connectedness	4.77 ± 1.233
Total CBE-24 score		4.74 ± 1.275

#### Level of nurses' perception about nurse caring behaviors

**Figure 2:**
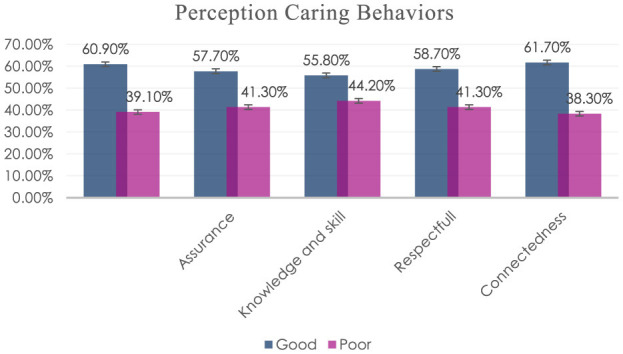
Perception of caring behaviors of nurses subscale in East Wallaga Zone Public Hospitals, Oromia, Ethiopia, 2023.

Among the subscales of caring behaviors, more than half (n = 219, 58.7%) of nurses had high “assurance of human presence,” (n = 208, 55.8%) of nurses had high “knowledge and skill,” (n = 219, 58.7%) of nurses had high “respectful deference to others,” and (n = 230, 61.7%) of nurses had high “positive Connectedness” (Figure 2).

Out of 373 respondents, more than half 228 (60.9%) (95% CI: 55.7–65.85) of nurses had a good perception of caring behaviors. This is supported by qualitative findings. For instance:

A 12 years of experience, Male nurse participant state that*, “My perception towards nurse care is good because nurses are very happy when we help patients and they get better. One thing that motivates me to have a positive outlook is that when a patient improves from a previous problem.”* (P2).

#### Bi-variable analysis of associated with nurses' perception of nurses caring behaviors

The research's findings indicate that the following variables were found to have a statistically significant association at (p < 0.25) with nurse perceptions of their caring behaviors: work experience (COR = 0.51, 95% CI 0.282–0.954), marital status (COR = 1.89, 95% CI 0.798–5.074), job satisfaction (COR = 3.090 95% CI 2.002–4.769), nurse-staff relationship (COR = 0.668 95% CI 0.440–1.014), workload (COR= 2.060 95% CI 1.337–3.174), and professional satisfaction (COR= 3.246 95% CI 2.099–5.018). However, there was no statistically significant correlation found between the perception of caring behaviors by nurses and age, sex, educational status, income, supervisor conflict, or stressful work environment (Table 5).

**Table 5:** Bi-variable analysis of factors associated with nurse perception about nurse caring behaviors in public hospitals of east Wollega zone, Oromia, Ethiopia, 2023.

**Variables**	**Category**	**Good perception (%)**	**Poor perception (%)**	**P-value**	**COR (95% CI)**
Age	20–25	27 (61.3%)	17 (38.6%)	0.815	1.121 (0.431–2.91)
	26–30	102 (57.9%)	74 (42.1%)	0.946	0.973 (0.438–2.16)
	31–35	52 (61.9)	32 (38%)	0.755	1.147 (0.485–2.711)
	36–40	29 (72.5%)	11 (27.5%)	0.230	1.86 (0.675–5.129)
	>40	17 (58.6%)	12 (41.3%)	1	1
Sex	Male	125 (63.4%)	72 (36/5%)	0.278	1.26 (0.83–1.911)
	Female	102 (57.9%)	74 (42.1%)	1	1
Marital status	Married	140 (63.34)	81 (36.6%)	0.064^*^	2.200 (0.954–5.074)
	Single	76 (59.8%)	51 (40.1%)	0.147^*^	1.897 (0.798–5.074)
	Widowed/divorced	11 (44%)	14 (56%)	1	1
Initial certificate they graduated	BSc degree	196 (62.2%)	119 (37.7%)	0.210^*^	1.435 (0.816–2.521)
	Diploma	31 (53.4%)	27 (46.5%)	1	1
Current educational level	Diploma	20 (58.8%)	14 (41.1%)	0.803	1.190 (0.303–4.682)
	Degree	201 (61.2%)	127 (38.7%)	0.653	1.319 (0.394–4.412)
	Masters	6 (54.5%)	5 (45.4%)	1	1
Work experience	1–5	89 (53.3%)	78 (46.7%)	0.035^*^	0.519 (0.282–0.954)
	6–10	94 (66.1%)	48 (33.8%)	0.718	0.890 (0.473–1.676)
	>10	44 (68.7%)	20 (31.2%)	1	1
Income	< 6,000	61 (58%)	44 (41.9%)	0.494	0.852 (0.538–1.349)
	>6,001	166 (61.9%)	102 (38%)	1	1
Hospital type	Primary	72 (66.6%)	36 (33.3%)	0.115	1.545 (0.899–2.657)
	General	18 (37.5%)	30 (62.5%)	0.029^*^	0.464 (0.233–0.924)
	Referral	71 (71%)	29 (29%)	0.027	1.892 (1.074–3.331)
	Specialized	66 (56.4)	51 (43.5)	1	1
	Medical	36 (51.4%)	34 (48.5)	0.279	0.454 (0.108–1.899)
	Surgical	43 (54.4%)	36 (45.5%)	0.356	0.512 (0.123–2.214)
	Pediatrics	34 (56.6%)	26 (43.3%)	0.432	0.560 (0.132–2.379)
Working unit	Emergency	34 (72.3%)	13 (27.6%)	0.881	1.121 (0.251–5.00)
	OPD	47 (64.3%)	26 (35.6%)	0.727	0.775 (0.185–3.253)
	ICU	18 (69.2%)	8 (30.7%)	0.964	0.964 (0.197–4.721)
	Orthopedic	8 (100%)	0	0.999	3.428 (0.567–1.453)
	Oncology	7 (70%)	3 (30%)	1	1
Job satisfaction	Satisfied	140 (73.6%)	50 (26.3%)	0.000^*^	3.090 (2.002–4.769)
	Dissatisfied	87 (47.5%)	96 (52.4%)	1	1
Conflict with supervisors	Conflict	114 (62.6%)	68 (37.3%)	0.492	1.157 (0.763–1.755)
	No conflict	113 (59.1%)	78 (40.8%)	1	1
Nurse-staff R/ship	Good R/ship	100 (55.8%)	79 (44.1%)	1	1
	Bad R/ship	127 (65.4%)	67 (34.5%)	0.058^*^	0.668 (0.440–1.014)
Stress full workplace	No stress	122 (58.6%)	86 (41.3%)	0.328	0.811 (0.532–1.234)
	Stress	105 (63.6%)	60 (36.3%)	1	1
Work load	Low	113 (53.5%)	98 (46.4%)	0.001^*^	0.485 (0.315–0.748)
	High	114 (70.3%)	48 (29.6%)	1	1
Professional satisfaction	Satisfied	141 (74.2%)	49 (25.7%)	0.000^*^	3.246 (2.099–5.018)
	Dissatisfied	86 (47%)	97 (53%)	1	1

“^*^p < 0.25” “1 = Reference Group.”

#### Factors associated with nurse perception of caring behaviors

All variables which showed statistical significance association with nurses' perception of nurse caring behaviors (*P* ≤ 0.25) in the bi-variable analysis were entered into a final logistic regression to avoid an excessive number of variables. After adjustment five variables are significantly associated with perception of caring behaviors (Table 6).

**Table 6:** Multi-variate analysis of factors associated with nurse perception about nurse caring behaviors in public hospitals of East Wollega zone, Oromia, Ethiopia, 2023.

**Variables**	**Category**	**Good perception (%)**	**Poor perception (%)**	**COR (95% CI)**	**AOR (95% CI)**	***P*-value**
Marital status	Married	140 (63.34%)	81 (36.6%)	2.200 (0.954–5.074)	2.69 (0.918–7.922)	0.071
	Single	76 (59.8%)	51 (40.1%)	1.897 (0.798–5.074)	3.90 (1.190–12.832)	**0.025** ^*^
	Widowed/divorced	11 (44%)	14 (56%)	1	1	1
Initial certificate they graduated	BSc degree	196 (62.2%)	119 (37.7%)	1.435 (0.816–2.521)	1.44 (0.216–1.930)	0.061
	Diploma	31 (53.4%)	27 (46.5%)	1	1	1
Work experience	1–5	89 (53.3%)	78 (46.7%)	0.519 (0.282–0.954)	0.43 (0.195–0.974)	**0.043** ^*^
	6–10	94 (66.1%)	48 (33.8%)	0.890 (0.473–1.676)	1.12 (0.528–2.398)	0.760
	>10	44 (68.7%)	20 (31.2%)	1	1	1
Hospital type	Primary	72 (66.6%)	36 (33.3%)	1.545 (0.899–2.657)	1.41 (0.729–2.746)	0.305
	General	18 (37.5%)	30 (62.5%)	0.464 (0.233–0.924)	0.59 (0.257–1.392)	0.233
	Referral	71 (71%)	29 (29%)	1.892 (1.074–3.331)	1.66 (0.835–3.332)	0.147
	Specialized	66 (56.4)	51 (43.5)	1	1	1
	Medical	36 (51.4%)	34 (48.5)	0.454 (0.108–1.899)	1.56 (0.765–3.213)	0.550
	Surgical	43 (54.4%)	36 (45.5%)	0.512 (0.123–2.214)	0.82 (0.386–1.763)	0.620
	Pediatrics	34 (56.6%)	26 (43.3%)	0.560 (0.132–2.379)	1.06 (0.471–2.427)	0.874
Working unit	Emergency	34 (72.3%)	13 (27.6%)	1.121 (0.251–5.00)	3.41 (1.353–8.626)	0.090
	OPD	47 (64.3%)	26 (35.6%)	0.775 (0.185–3.253)	1.17 (0.541–2.565)	0.679
	ICU	18 (69.2%)	8 (30.7%)	0.964 (0.197–4.721)	2.73 (0.870–8.582)	0.085
	Orthopedic	8 (100%)	0	0.151 (0.567–1.453)	0.73 (0.001–1.347)	0.999
	Oncology	7 (70%)	3 (30%)	1	1	1
Job satisfaction	Satisfied	140 (73.6%)	50 (26.3%)	3.090 (2.002–4.769)	2.47 (1.468–4.178)	**0.001** ^*^
	Dissatisfied	87 (47.5%)	96 (52.4%)	1	1	1
Nurse-staff R/ship	Good R/ship	100 (55.8%)	79 (44.1%)	1	1	1
	Bad R/ship	127 (65.4%)	67 (34.5%)	0.668 (0.440–1.014)	0.044 (1.210–3.451)	**0.007** ^*^
Work load	Low	113 (53.5%)	98 (46.4%)	0.485 (0.315–0.748)	0.65 (0.381–1.108)	0.113
	High	114 (70.3%)	48 (29.6%)	1	1	1
Professional satisfaction	Satisfied	141 (74.2%)	49 (25.7%)	3.246 (2.099–5.018)	3.38 (1.965–5.844)	**0.000** ^*^
	Dissatisfied	86 (47%)	97 (53%)	1	1	1

“^*^p < 0.05” “1 = Reference Group”. Bold values indicate significant variables.

In this particular study, Nurses who had single were 3.9 times more likely to perceive good caring behavior than compared to those who were divorced and widowed [AOR 95% CI 3.90 (1.190–12.832)]. Nurses who have had worker longer-duration work experience in their profession were more likely to perceive good caring behaviors as compared to lesser working experience. Nurses who had 1–5 years of experience were 0.43 times less likely to highly perceive good caring behavior than those compared with nurses who had more than 10 years of experience [AOR 95% CI 0.43 (0.195–0.974)].

Among Staff related factors, Nurses who satisfied with their job were 2.4 times more likely to perceive a good perception of caring behaviors compared to those who dissatisfied with their job [AOR 95% CI 2.47 (1.468–4.178)]. This is supported by qualitative findings. For instance:

A participant with 10 years experiences stated that, “*I have a good perception because nurses are very happy when they help patients and they get better. One thing that motivates me to have a positive outlook is that when a patient improves from a previous problem and shows positive changes, your heart relaxes.”* (P2), *(*P3).

Nurses who were satisfied with the profession were 3.3 times more likely to perceive good caring behaviors compared to nurses who were unsatisfied [AOR 95% CI 3.38 (1.965–5.844)]. This is confirmed by qualitative findings. For instance:

From participants with 13 years of experiences confirmed that, “*What is nursing care for patients, the continuous services provided by the nurse to the sick patient who comes to the health facility, when they are sick, especially at the hospital level patients come and most of them are treated as inpatients.”* (P5).

Other participants with 12 years of experiences confirmed that “*When you give care, if you prescribe medicine for a patient now, you don't know if he is cured or not, somehow pain accuses you of pain, he says he's wearing it, so at hand, you see the actual thing in your hands. It's something tangible. (P1)”*

Nurses who have bad nurse-staff relationship were 0.04 less likely to highly perceive good caring behaviors than compared to nurses who have good relationship with their staff. [AOR, 95% CI, 0.044 (1.210–3.451)].

This is confirmed by qualitative findings. For instance:

A participant with 12 years of experiences stated by sharing his experience that*, “The other side of the pressure is there is ignorance, which ignores this profession, why there is ignorance one of the care you give is usually nursing care, they refer you to medical care, from the management, from the doctor you work with.”* (P1).

### Findings and emerged themes

Analysis of the interviews data initially led to construction of 50 codes. But after repeated data analysis, subsequent deletion of duplicate codes and merging similar items. Thirteen main themes and thirty sub-themes or categories that come from data analysis (Table 7).

**Table 7:** Extracted themes and sub-themes regarding level of perception on nurse caring behaviors among nurses working in EWZ Public Hospitals, Nekemte, Ethiopia, 2023.

**Themes**	**Sub-themes**
**Identified theme regarding level of perception**	
Good perception	Nursing care is holistic care
	Happiness of Nurses when they help patients and get pleasure from nursing care
	Issue of better understanding of nursing care behavior
	Issue of motivation when a patient improves from a previous problem and shows positive changes
Poor perception	Issue of attitude toward nursing profession
	Issue of dominance on nursing professionals
**Identified themes regarding hospital-environment**	
Work load	Issue of potential pressures on nurse delivery of care
	Expected patient ratio to nurse ration
Stressful work place	Environment of working unit for the procedure
	Structure and setup of hospitals with their stations
	Sterility of the room for doing nursing interventions
Nurse-staff relationship	Pressure from professionals in other fields
	Ignorance and pressure from managements and professionals in other fields
Conflict with supervisors	Managements misunderstanding through their leadership
	Aspect of leadership and supervisors
Env't status/security issue	Mass-quasility due to Accident
	Personal protective equipment is not enough during mass-quasiality
**Identified themes regarding job and professional satisfaction**	
Job satisfaction	Nurses are happy with their job when they help patients and they get better.
	There is a lack of knowledge gap on applying the procedure
	Giving various care for the patients during interventions
	Payment, incentives and allowances
Professional satisfaction	Nursing is independent profession
	Care Nursing profession is continuous
**Identified themes regarding endemic disease**	
Suddenly arise disease	Suddenly arise disease
**Identified themes regarding improvement of perception of NCB**	
Changing attitude	Attitude toward our profession should be changed
Educational opportunity and training	Giving trainings related with the profession
	Expand chance of education related to nursing
	Nursing standard of care and Job description must be improved
Encouragement and motivating nurses	Encouraging and motivating nurses by finance, training, promotion
	First motivation to improve the nursing profession

### Issues related with good perception

#### Nursing care is holistic care, it needs better understanding

The majority of participants stated that nursing care is the main part of the nursing profession and majority of them have good perception of nurse to caring behavior. An EOPD male nurse with 12 years of experience stated that, “*Nursing care is holistic a*n*d Nurse profession is focused on care rather than medicine. They look at disease-focused, but nurses look at the patient's physiologic needs based on what the patient going through now, based on what he/she is suffering from now, and giving him/her care.”* (P1). Again participants of 29 years old with 6 years of experience confirm that, “*The main tasks for the topic are what people come looking for, what people expect from us, it is care.”* (P6). The participants of the study reported that, they have better understanding of nurse caring behaviors. A participant of 34 age and 13.5 years of experiences confirm that*, “What is nursing care for patients is the continuous services provided by the nurse to the sick patient who comes to the health facility, So I have a good perception towards a better understanding of nursing care behavior.”* (P5).

#### Identified themes regarding hospital-environment related factors

Five main themes emerged under this hospital-Environment related issues and have their own sub-themes. Good Perception of caring behaviors among nurses well, if the Environment of the hospital is safe and good for professionals. If the hospitals environment is not appropriate for health professionals there is no good care for the patients.

### Issues of work load

#### Potential pressures on nurse and expected patient to nurse ratio

Nursing work is complex and involves several aspects of practice that are common to the profession which can generate high workloads. A participants with 10 and 12 years of experience, respectively, stated that, “*One of the is due to workload, as the relationship or estimate of the nurse with a patient does not match the ratio and the load on the nurse increases care may decline. As a standard, a nurse has to treat that many patients and it doesn't apply whether it's the FMOH or the WHO who puts it down.”* (P1) (P2). From some of our respondents, this high work load affects the perception of nurses during care. Another participant with 2 years of experiences confirmed that, “*There is a work load so far although it isn't much it doesn't make an impact either. There is a patient ratio to nurse ration and that is not expected.”* (P4).

### Issues of nurse-staff relation ship

#### Ignorance, pressure from managements and professionals in other fields

The nurse-staff relationship has expressed the importance of having an environment in which nursing colleagues co-operated and support one another in clinical situations to achieve the best possible care for patients in need. But there are obstacles between individual nurses and other staff. And this affects Perception of nurses. A participant with 12 years of experiences stated by sharing his experience that*, “The other side of the pressure is there is ignorance, which ignores this profession, why there is ignorance one of the care you give is usually nursing care, they refer you to medical care, from the management, from the doctor you work with.”* (P1). Another participant with 2 years of experience confirmed that, “*Occasionally, there are times when there is occasional pressure from professionals in other fields.”* (P4).

### Issues of conflict with supervisors/management

#### Managements, leadership and supervisors miss-understanding

Some respondents suggested that a good supervisor or good manager was an important factor in achieving organizational flexibility and handling those health professions. Respondents who were working in clinical units saw significant organizational changes as well as professional changes had been implemented, suggested that the hospital manager, supervisors, and ward manager roles were crucial to the success of these changes. A participant with 12 years of experience stated that, “*For example, what kind of impact is created by management there is a misunderstanding through their leadership. Management from top to bottom fails to understand nurses as not a good assistant to help physicians.”* (P1).

Other respondents suggested, however, that the lack of a manager and supervisor had failed to change the organization as well as the perception of caring behavior of the nurse professionals. As 25 years old and 2 years of working experiences confirmed that, “*The occasional aspect of leadership is nothing. Occasionally, sometimes people come to training and gather us together and give us little bits of information. Other than that, there is pressure in that regard*.” (P4).

### Issues of environmental security or safety

#### Mass-quality and lack of PPE during procedure

Some of our participants display their ideas, a participant with 13 years of experiences stated that, “*It causes a lot of pressure, for example when we talk about security issues when different parties clash, there can be mass-quality between the parties or the most injured person can be hit, then with all the people who came injured to reach you are just.”* (P5).

From some participants these also affects caring behaviors. As participant with 10 and 12 years of experience says respectively, “*Yes, it does affect. For example, if you take it as an emergency when a problem occurs, if the patient is injured, displaced, and comes with various problems, the load will increase and the care he provides may decrease, why because personal protective equipment is not enough in our country.” (P2) (P1)*.

### Issues of job satisfaction

#### Nurses are happy with their job and they give various care for the patients

When nurses are satisfied with their jobs, they tend to display more caring behaviors, thereby contributing to the achievement of organizational goals related to the provision of quality healthcare services. This suggests that the nurses had been providing quality care and acting in the best interests of their patients. A participant with 10 years experiences stated that, “*I have a good perception because nurses are very happy when we help patients and they get better, your heart relaxes and it makes me have a positive outlook.”* (P2). A Participant with 6 years of experience confirmed “*We can give various care, we can raise vital signs, give prescribed medication…it is our role to follow the prescribed medication legally. Not only that, rolling patients from time to time, checking patients from time to time, even removing bedridden patients from time to time, giving various oral care mouth care are great jobs.”* (P6).

### Issues professional satisfaction

#### Nursing care is independent and continuous

“*When you give care, if you prescribe medicine for a patient now, you don't know if he is cured or not, you take him home, but when you give care, someone comes bleeding you, you lose blood. so at hand, you see the actual thing in your hands. It's something tangible (Participant 1, aged 37, male, year 12)”* From participants with 13 years of experiences confirmed that, “*What is nursing care for patients, the continuous services provided by the nurse to the sick patient who comes to the health facility, when they are sick, especially at the hospital level patients come and most of them are treated as inpatients, which is continuous nursing they want that care.”* (P5).

### Identified themes regarding endemic disease

#### Suddenly arise diseases

Working during a pandemic or endemic is a unique situation and it is imperative to be aware of its special characteristics. There is also work overload and stress related to this during sudden problems. It is known nurses are concerned with their health and personal safety, their families' health, and the need to communicate with team members and family rather than having good care behaviors. This affects the quality of care also the perception of having good caring behaviors among nurse professionals. A participant with 6 years of experiences stated that, “*Yes, it can have a huge impact. If any disease arises, one may overburden by disease, the workload may also occur and if a professional is sick if I am infected the person I treated may be affected for example he may stop getting treatment, which can cause various pressures.”* (P6).

## Discussion

This study determined the perception of caring behavior and associated factors among nurses working in east Wollega zone public hospitals. According to the result of all dimensions of CBI showed that 60.9% with CI: (55.7–65.8) had a good perception of caring behaviors. A relatively high proportion of nurses had positive connectedness (61.7%) followed by patient respectfulness (58.7%), assurance of human presence (57.8%), and professional knowledge and skill (55.8%).

This result is supported by the result obtained from interview, mentioned by A 12 years of experience, Male nurse participant state that*, “My perception towards nurse care is good because nurses are very happy when we help patients and they get better, your heart relaxes and it makes me have a positive outlook.”* Then majority of participants have good perception on caring behaviors. This finding is supported by qualitative study done at Taiwanese nurses, they have positive outlook on perception of caring behaviors (28).

This result is in line with research from hospitals in Harari City, where 63.4% of nurses (CI: 58.5–68.2%) thought well of the caring behaviors they observed (12). However, this finding was greater than research from public hospitals in the Harar region, where 51% of nurses thought well of the good caring behaviors they observed (19). This discrepancy may be explained by variations in the study's sample size, timing, and participants all of which are limited to inpatient nursing professionals. They also only utilize two hospitals. It might be the instrument used to assess the difference in the caring behaviors component. This study's finding is also lower than the study by Jimma and Gonder, the proportion of nurses who had a high perception of caring behaviors was found to be 80.4%, 68.2%, respectively (11, 18). And also lower than study conducted in north Iran to investigate nurses' perceptions of caring behaviors and related factors in cardiac intensive care units of Gilan University of Medical Sciences, it was found that nurses' mean perceptions of caring behaviors were about 81.8% (16). This difference may be due to organizational nature, study setting, and sample size. More they select only referral hospitals and higher hospitals. They don't include primary and general hospitals.

The study finding showed that the Mean of the total CBI-16 score of nurses' perception of nurse caring behaviors in four sub-scores was found to be 4.74. This finding is much higher than the study done at Harari Town which is the total score of CBI-24 I four Subscale was 4.21 ± 1.08 (12). But a lower total score of CBI-24 in four sub-scales than the result of the Study done in Turkish and Greek by Mean ± SD (5.28 ± 0.44) and (4.95 ± 0.72), respectively (29–31). This all might be due to differences in the tool we use to measure CBI-16 and CBI-24. I use the standardized and latest which have 16 components subscales. Others are due to differences in curriculum and training of nurses, working environment, socio-economic condition as well and development level of the countries.

This study indicated that Marital Status, work experience, Job satisfaction, nurse-staff relationship, and professional satisfaction were all significantly associated with the perception of caring behavior. The odds of having a good perception of caring behavior were 3.9 times more likely among nurses who are single compared to those who were divorced and widowed. This finding was in line with the findings of the Harari town study and the Greek study (12, 32). The reason might be that single persons take/feel more responsibility, are more stable in life, motivated and happy in their life so they concentrate on their profession and their job.

Compared to nurses with less work experience, those with longer work experience were more likely to perceive good caring behaviors in their career. Compared to nurses with high levels of professional work experience, those with 1–5 years of experience were 0.43 times less likely to highly perceive caring behaviors. This result is consistent with a Gonder study of nurses who thought they were exhibiting caring behaviors (11). The reason might be the longer work experience, the most understanding of the nursing profession's value, and increased the maturity level that becomes an expert level in nursing practice. Moreover, work experience is very important for every profession, especially in nursing.

Nurses who had professional satisfaction with the nursing work were 3.3 more likely to highly perceived caring behaviors compared to professional unsatisfied nurses. This result is supported by other studies done in different areas (11, 12, 17–19). The reason could be professional values and identity of nursing which may influence the perception of caring behaviors. Moreover, a professional practice characteristic may be greatly led to job satisfaction that may improve their perception of professional caring behaviors. This result is supported by the result obtained from interview mentioned by A 12 years of experiences confirmed that “*When you give care, if you prescribe medicine for a patient now, you don't know if he is cured or not, you take him home, but when you give care, you see the actual thing in your hands. It's something tangible.”*

Nurses who satisfied with their job were 2.4 times more likely to perceive a good perception of caring behaviors compared to those who dissatisfied with their job. This result is supported by other studies done in different areas (11, 12, 17). One possible explanation is that nurses' perceptions of caring actions are influenced by job satisfaction, which increases their incentive to provide high-quality care and act in their patients' best interests (33, 34). This result is supported by result obtained from interview mentioned that, A 4 years of experiences confirmed that, “*What you or the care you do is for the patient or the sick person needs care, the critical patient may come, there is a center coming and on this, I will provide full service, is sick care isn't it? So I'm always ready to give my patient the necessary care.”*

From findings of previous study, when nurses are satisfied with their jobs, they tend to display more perceived caring behaviors, thereby contributing to the achievement of organizational goals related to the provision of quality healthcare services. The emergent link between job satisfaction and nurse's caring behaviors is consistent with the results of past studies also support this idea (18).

Nurses who have bad nurse-staff relationship were 0.04 less likely to highly perceive good caring behaviors than compared to nurses who have good relationship with their staff. A possible explanation for this finding is poor working relationships between nurses and other coworkers may have an impact on creating a healthy work atmosphere and giving nurses more freedom to express their professional concern for the quality of care. If there is no good relationship among staffs there is no good caring behaviors, because co-operative and working together is crucial among health workers. Because the profession is holistic and interpersonal (35). This result is supported by result obtained from interview mentioned that, A participant with 12 years of experiences stated by sharing his experience that*, “The other side of the pressure is there is ignorance, which ignores this profession, why there is ignorance one of the care you give is usually nursing care, they refer you to medical care, from the management, from the doctor you work with.”*

This finding in line with qualitative study at Hong Kong, also revealed that, the nurse-staff relationship has expressed the importance of having an environment in which nursing colleagues co-operated and support one another in clinical situations to achieve the best possible care for patients in need. But there are obstacles between individual nurses and other staff (35).

Working during a pandemic or endemic and issue of environmental safety or security is a unique situation and it is imperative to be aware of its special characteristics. There is also work overload and stress related to this during sudden problems and unsafe environments. It is known nurses are concerned with their health and personal safety, their families' health, and the need to communicate with team members and family rather than having good care behaviors. This affects the quality of care also the perception of having good caring behaviors among nurse professionals. A participant with 6 years of experiences stated that, “*Yes, it can have a huge impact. If any disease arises, one may overburden by disease, the workload may also occur and if a professional is sick if I am infected the person I treated may be affected for example he may stop getting treatment, which can cause various pressures.”* (P6).

### Strengths

- Using a standardized, latest, and internationally acceptable tools (CBI-16)
- The investigation includes all levels of hospital nurse professions (primary, General, Referral, and Specialized hospitals) and multi-centered, which increases generalizability.
- Selecting study design and adding qualitative study for more finding and reducing self-reported idea to maximize the reliability of data.

### Limitation

- The study only showed situations at one point in time, it did not show cause and effect relationships.
- There might be respondent bias due to self-reported questionnaires in the quantitative part.
- The study only stated one side, mean that nurses perception.

## Conclusion and recommendation

### Conclusion

The proportion of having the good perception of caring behaviors among nurses was found to be low relative to literature. In this study, marital status, work experience, job satisfaction, professional satisfaction, and nurse-staff relationship were found to be the associated factors of the perception of nurses toward caring behaviors. Therefore, perception of nurses caring behaviors can be enhanced by creating conductive nurse staff relationships and making nursing active in their work to improve nurse's perception toward caring behaviors.

### Recommendations

- Nurse directors, supervisors, and ward heads need to motivate nurses for:

° Returning to the patients voluntarily to provide nursing care timely and encourage the patient to call if there are problems.
° Talking with the patients
° Responding quickly to the patient's calls, because all of the Assurance of human presence sub-scale components in CBI according to this study are low.

- Hospital management and supervisors need to create harmonized work relations, providing equipment for work, creating a good relationship among staff, providing training, and upgrading education for nurses to improve perception of nurses on caring behaviors.
- East Wollega Zone Health Bureau need to apply strong relationship with their nurse professionals and follow their problems, accepting and respecting their questions and supportive supervision of hospitals.
- Nursing institutions, Health bureaus, and ENA, need to pay attention to quality nurse care and to improve perception of nurses on caring behaviors.
- Further research on nurse's perception of caring behaviors using different study design need to be done. Especially Comparative study design which focus on nurses and patients perception. Due to this specific research is focus only nurses side aspect.

## Data Availability

The raw data supporting the conclusions of this article will be made available by the authors, without undue reservation.

## References

[1] Watson J. Unitary Caring Science: Philosophy and Praxis of Nursing. University Press of Colorado (2018).

[2] Bucco T. The Relationships Between Patients' Perceptions of Nurse Caring Behaviors, Nurses' Perceptions of Nurse Caring Behaviors and Patient Satisfaction in the Emergency Department. Seton Hall University (2015).

[3] Azizi-Fini I Mousavi M-S Mazroui-Sabdani A Adib-Hajbaghery M. Correlation between nurses' caring behaviors and patients' satisfaction. Nurs Midwifery Stud. (2012) 1:36–40. 10.5812/nms.7901

[4] Abou Elnaga A. The impact of perception on work behavior. Kuwait Chap Arab J Bus Manag Rev. (2012) 2:1–16.

[5] Jarrar Mt Minai MS Al-Bsheish M Meri A Jaber M. Hospital nurse shift length, patient-centered care, and the perceived quality and patient safety. Int. J. Health Plann. Manag. (2019) 34:e387–96. 10.1002/hpm.2656 30221794

[6] Gishu T Weldetsadik AY Tekleab AM. Patients' perception of quality of nursing care; a tertiary center experience from Ethiopia. BMC Nurs. (2019) 18:1–6. 10.1186/s12912-019-0361-z 31427889 PMC6694623

[7] Gaudenz C De Geest S Schwendimann R Zúñiga F. Factors associated with care workers' intention to leave employment in nursing homes: a secondary data analysis of the Swiss Nursing Homes Human Resources Project. J Appl Gerontol. (2019) 38:1537–63. 10.1177/0733464817721111 28715925

[8] Chang S Yang W Deguchi H. Care providers, access to care, and the long-term care nursing insurance in China: an agent-based simulation. Soc Sci Med. (2020) 244:112667. 10.1016/j.socscimed.2019.112667 31734601

[9] Thomas D Newcomb P Fusco P. Perception of caring among patients and nurses. J Pat Exp. (2019) 6:194–200. 10.1177/2374373518795713 31535007 PMC6739676

[10] Youssef H Mansour M Ayasreh I Al-Mawajdeh N. A medical-surgical nurse's perceptions of caring behaviors among hospitals in Taif city. Life Sci J. (2013) 10:720–30.

[11] Ashenafie TD Tebeje NB Gardew DG Gebrie MH. Perception of caring behaviours and associated factors among nurses working in Gondar university and felege-hiwot referral hospitals, northwest Ethiopia: a cross-sectional study. Int J Nurs Sci. (2020) 9:490–5.

[12] Fikre A Egata G Abdisa L Yadeta E Eyeberu A Dheresa M. Perception of caring behaviors and associated factors among nurses working in Harar Hospitals, Eastern Ethiopia. SAGE Open Nurs. (2022) 8:23779608221143909. 10.1177/23779608221143909 36569513 PMC9768825

[13] Salimi S Azimpour A. Determinants of nurses' caring behaviors (DNCB): preliminary validation of a scale. J Car Sci. (2013) 2:269. 25276735 10.5681/jcs.2013.032 PMC4134150

[14] Vandenhouten C Kubsch S Peterson M Murdock J Lehrer L. Watson's theory of transpersonal caring: Factors impacting nurses professional caring. Holist Nurs Pract. (2012) 26:326–34. 10.1097/HNP.0b013e31826ed0e8 23075749

[15] Pasinringi S Wandy I Fakianti A Ameliyah A. The level of patient satisfaction with hospital services under national health insurance program Inmakassar City, Indonesia. Int J Health Sci. (2015) 8.

[16] Moghimi ES Lotfabad LS Samani NK Kargar L Chafjiri AS Leily EK. The nurses' perceptions of caring behaviors and related factors in cardiac intensive care units in the north of Iran. Arch Venezolanos Farmacol Terapéutica. (2022) 41:110–20.

[17] Assefa A Getahun D Desalegn N Kefelew E Molla W Assefa DG. Perception of caring behavior and associated factors among nurses and midwives working in public hospitals in Southern Ethiopia. Int J Nurs Sci. (2022) 9:490–5. 10.1016/j.ijnss.2022.09.014 36285090 PMC9587403

[18] Oluma A Abadiga M. Caring behavior and associated factors among nurses working in Jimma University specialized hospital, Oromia, Southwest Ethiopia, 2019. BMC Nurs. (2020) 19:19. 10.1186/s12912-020-0407-2 32210736 PMC7087356

[19] Kibret H Tadesse B Debella A Degefa M Regassa LD. Level and predictors of nurse caring behaviors among nurses serving in inpatient departments in public hospitals in Harari region, eastern Ethiopia. BMC Nurs. (2022) 21:76. 10.1186/s12912-022-00856-8 35365137 PMC8972678

[20] FDRE. Federal Democratic Republic of Ethiopia. Compassionate, Respectful and Caring (CRC) Health Workforce (2017).

[21] Ferede AJ Erlandsson K Gezie LD Geda B Wettergren L. Psychometric properties of the caring behaviors inventory-16 in Ethiopia. Nursing Reports. (2022) 12:387–96. 10.3390/nursrep12020037 35736614 PMC9229844

[22] Alikari V Fradelos EC Papastavrou E Alikakou S Zyga S. Psychometric properties of the Greek version of the caring behaviors inventory-16. Cureus. (2021) 13:e15186. 10.7759/cureus.15186 34178507 PMC8221654

[23] Heritage B Pollock C Roberts LD. Confirmatory factor analysis of Warr, Cook, and Wall's (1979) Job satisfaction scale. Aust Psychol. (2015) 50:122–9. 10.1111/ap.12103

[24] Liao C Qin Y He Y Guo Y. The nurse–nurse collaboration behavior scale: development and psychometric testing. Int J Nurs Sci. (2015) 2:334–9. 10.1016/j.ijnss.2015.10.005

[25] Zaghloul AA. Developing and validating a tool to assess nurse stress. J Egypt Public Health Assoc. (2008) 83:223–7. 19302776

[26] Neill D Davis GC. Development of a subjective workload assessment for nurses: a human factors approach. J Nurs Meas. (2015) 23:452–73. 10.1891/1061-3749.23.3.452 26673770

[27] Lynn MR Morgan JC Moore KA. Development and testing of the satisfaction in nursing scale. Nurs Res. (2009) 58:166–74. 10.1097/NNR.0b013e3181a308ba 19448520

[28] Tsai Y-C Wang Y-H. Caring behavior exhibited by Taiwanese nurses. Int J Car Sci. (2015) 8:317.

[29] Erkus G Dinc L. Turkish nurses' perceptions of professional values. J Prof Nurs. (2018) 34:226–32. 10.1016/j.profnurs.2017.07.011 29929805

[30] Kiliç M Öztunç G. Comparison of nursing care perceptions between patients who had surgical operation and nurses who provided care to those patients. Int J Car Sci. (2015) 8:625.

[31] Papastavrou E Efstathiou G Tsangari H Suhonen R Leino-Kilpi H Patiraki E. Patients' and nurses' perceptions of respect and human presence through caring behaviours: a comparative study. Nurs Ethics. (2012) 19:369–79. 10.1177/0969733011436027 22581506

[32] Karlou C Papathanassoglou E Patiraki E. Caring behaviours in cancer care in Greece. Comparison of patients', their caregivers' and nurses' perceptions. Eur J Oncol Nurs. (2015) 19:244–50. 10.1016/j.ejon.2014.11.005 25648496

[33] De Los Santos JAA Labrague LJ. Job engagement and satisfaction are associated with nurse caring behaviours: a cross-sectional study. J Nurs Manag. (2021) 29:2234–42. 10.1111/jonm.13384 34021940

[34] Akinwale OE George OJ. Work environment and job satisfaction among nurses in government tertiary hospitals in Nigeria. Rajagiri Manag J. (2020) 14:71–92. 10.1108/RAMJ-01-2020-0002

[35] Yam B Rossiter JC. Caring in nursing: perceptions of Hong Kong nurses. J Clin Nurs. (2000) 9:293–302. 10.1046/j.1365-2702.2000.00349.x 11111621

[36] Warr P Cook J Wall T. Scales for the measurement of some work attitudes and aspects of psychological well-being. J Occup Psychol. (1979) 52:129–48. 10.1111/j.2044-8325.1979.tb00448.x

